# Preventing Obesity in the Military Community (POMC): The Development of a Clinical Trials Research Network

**DOI:** 10.3390/ijerph120201174

**Published:** 2015-01-22

**Authors:** Elena A. Spieker, Tracy Sbrocco, Kelly R. Theim, Douglas Maurer, Dawn Johnson, Edny Bryant, Jennifer L. Bakalar, Natasha A. Schvey, Rachel Ress, Dean Seehusen, David A. Klein, Eric Stice, Jack A. Yanovski, Linda Chan, Shari Gentry, Carol Ellsworth, Joanne W. Hill, Marian Tanofsky-Kraff, Mark B. Stephens

**Affiliations:** 1 Department of Family Medicine, Madigan Army Medical Center, 9040 Fitzsimmons Avenue, Joint Base Lewis-McChord, Tacoma, WA 98431, USA; E-Mails: elena.a.spieker.ctr@mail.mil (E.A.S.); douglas.m.maurer.mil@mail.mil (D.M.); 2 Department of Medical and Clinical Psychology, Uniformed Services University of the Health Sciences, 4301 Jones Bridge Road, Bethesda, MD 20814, USA; E-Mails: kelly.theim.ctr@usuhs.edu (K.R.T.); dawn.johnson@usuhs.edu (D.J.); edny.joseph@usuhs.edu (E.B.); Jennifer.bakalar@usuhs.edu (J.L.B.); natasha.schvey.ctr@usuhs.edu (N.A.S.); rachel.ress.ctr@usuhs.edu (R.R.); marian.tanofsky-kraff@usuhs.edu (M.T.-K.); 3 The Henry M. Jackson Foundation for the Advancement of Military Medicine, Inc., 6720A Rockledge Dr., Bethesda, MD 20817, USA; 4 Department of Family and Community Medicine, Nelson Hall, Fort Gordon, GA 30905, USA; E-Mail: Dean.A.Seehusen.mil@health.mil; 5 Department of Family Medicine, Fort Belvoir Community Hospital, 9300 DeWitt Loop, Fort Belvoir, VA 22060, USA; E-Mail: david.a.klein26.mil@mail.mil; 6 Oregon Research Institute, 1776 Millrace Dr., Eugene, OR 97403, USA; E-Mail: estice@ori.org; 7 Section on Growth and Obesity, Program in Developmental Endocrinology and Genetics, *Eunice Kennedy Shriver* National Institute of Child Health and Human Development, National Institutes of Health, DHHS, Bethesda, MD 20892, USA; E-Mail: yanovskj@mail.nih.gov; 8 Department of Obstetrics and Gynecology, Naval Hospital Camp Lejeune, 100 Brewster Blvd., Camp Lejeune, NC 28547, USA; E-Mail: Linda.Chan@med.navy.mil; 9 Department of Family Medicine, Naval Hospital Camp Lejeune, 100 Brewster Blvd., Camp Lejeune, NC 28547, USA; E-Mails: shari.gentry@med.navy.mil (S.G.); carol.ellsworth@med.navy.mil (C.E.); 10 Department of Research, Naval Hospital Camp Lejeune, 100 Brewster Blvd., Camp Lejeune, NC 28547, USA; E-Mail: Joanne.Hill@med.navy.mil; 11 Department of Family Medicine, Uniformed Services University of the Health Sciences, 4301 Jones Bridge Road, Bethesda, MD 20814, USA; E-Mail: mark.stephens@usuhs.edu

**Keywords:** patient centered medical home, military, weight gain, obesity, prevention, pregnancy, early career, loss of control eating, adolescence

## Abstract

Obesity impacts the U.S. military by affecting the health and readiness of active duty service members and their families. Preventing Obesity in Military Communities (POMC) is a comprehensive research program within Patient Centered Medical Homes (PCMHs) in three Military Training Facilities. This paper describes three pilot randomized controlled trials that target critical high risk periods for unhealthy weight gain from birth to young adulthood: (1) pregnancy and early infancy (POMC-Mother-Baby), (2) adolescence (POMC-Adolescent), and (3) the first tour of duty after boot camp (POMC-Early Career). Each study employs a two-group randomized treatment or prevention program with follow up. POMC offers a unique opportunity to bring together research and clinical expertise in obesity prevention to develop state-of-the-art programs within PCMHs in Military Training Facilities. This research builds on existing infrastructure that is expected to have immediate clinical benefits to DoD and far-reaching potential for ongoing collaborative work. POMC may offer an economical approach for widespread obesity prevention, from conception to young adulthood, in the U.S. military as well as in civilian communities.

## 1. Introduction

### 1.1. Obesity in the U.S. Military

More than one-third of the U.S. adult population is obese [1]. The number of children and adolescents who are either overweight (body mass index [BMI], kg/m^2^ ≥ 85th < 95th percentile) or obese (BMI ≥ 95th percentile) has tripled since 1970 [1] and rates among dependent children [2] nearly parallel rates among civilian children. Despite standard fitness requirements, more military personnel are overweight than civilians, but obesity rates remain higher among civilians [2]. An estimated 54% of men and 34% of women serving on active duty are overweight, and 12% of active duty personnel are obese [3], represented across all services [3]. Military children, particularly boys, are more likely than their civilian peers to join the military [4]. Compared to civilian children by age and gender, military child dependents have lower rates of overweight and obesity [2]. Among dependents, 2012 rates of overweight and obesity were highest among 12–17 year old male (15.2%, 15.0%) and female (17.1%, 12.4%) dependents [2]. This is concerning since the risk of an overweight child or adolescent becoming obese as an adult increases with age and degree of overweight [5]. The resulting personal cost and the impact on the Department of Defense (DoD) is high, as dependent children are at high risk for impairments in metabolic functioning [6], Type 2 diabetes [7], and continued obesity in adulthood [8,9].

### 1.2. Impact of obesity within the DoD: Costs and Security

In addition to the economic costs of obesity and related co-morbidities, which are estimated to exceed one billion annually [10], excess weight may be particularly costly to the DoD as obesity significantly impacts readiness and national defense. Effects of excess weight may include higher absenteeism, fewer eligible military recruits, and decreased retention. These weight-related costs not only influence U.S. military readiness but may also threaten national security. The federal health care system also incurs obesity-related financial burdens past retirement or discharge from the military through Veteran’s Administration (VA) medical benefits. Over three-quarters (78%) of veterans are overweight or obese [11] with veterans returning from deployment at particularly high risk, especially men with PTSD and women diagnosed with depression [12]. The VA has addressed issues with weight by implementing programs such as MOVE!, but with low overall estimates of participation and preliminary effectiveness [13], more is needed in preparation for the next generation.

In addition to caring for active duty service personnel, the DoD is also responsible for healthcare provided to military spouses and children (dependents). Military dependents, particularly those of recently deployed personnel, are at elevated risk for difficulties in psychological functioning [14,15,16]. Although the data are sparse, eating disturbances appear to be one of the most common co-morbid psychological problems identified in adolescent dependents [17]. Furthermore, pre-pregnancy obesity, excess weight gain during pregnancy, and children who experience rapid and excess growth during the first year of life are at high-risk for subsequent obesity [5]. Taken together, preventing excess weight gain in service members, veterans, and dependents could potentially alleviate serious problems related to obesity and significantly reduce costs for both the DoD and VA.

Despite the fact that a large number of individual programs and interventions currently exist, there remains a need to identify actions for weight gain prevention that are capable of creating lasting change at the societal level. The Institute of Medicine’s Committee on Accelerating Progress in Obesity Prevention has noted that designing more effective obesity prevention programs is a public health priority. To address those at greatest risk for obesity, we created a multi-faceted preventive collaboration, Preventing Obesity in Military Communities (POMC). This innovative program targets obesity from conception to young adulthood within the U.S. military family.

### 1.3. Objectives of POMC

The overall aim of the POMC project is to create a synergistic obesity prevention program within the PCMH for three high-risk periods (pregnancy, adolescence, and early career military). A secondary aim is to utilize the existing PCMH structure used in military treatment facilities within the Military Primary Care Research Network (MPCRN) to strengthen and build the capacity of this network to conduct further research on weight-related issues in the military. The MPCRN represents one of the largest practice-based research networks in the country that connects 17 Family Medicine residency training sites throughout the DoD and includes 600,000 eligible DoD beneficiaries eligible for care. Each of the 17 MPCRN sites also houses a Family Medicine residency for post-graduate medical education. This represents a robust nucleus of over 400 physicians in training who are supported by over 1000 faculty and support personnel. For the proposed program, prevention projects will be evaluated at three MCPRN member sites, which were intentionally selected due to their training missions, patient demographics and leadership experience. Military site PIs for the project are all family medicine physicians and active duty military officers.

## 2. Experimental Section

### 2.1. Overview

**Figure 1:**
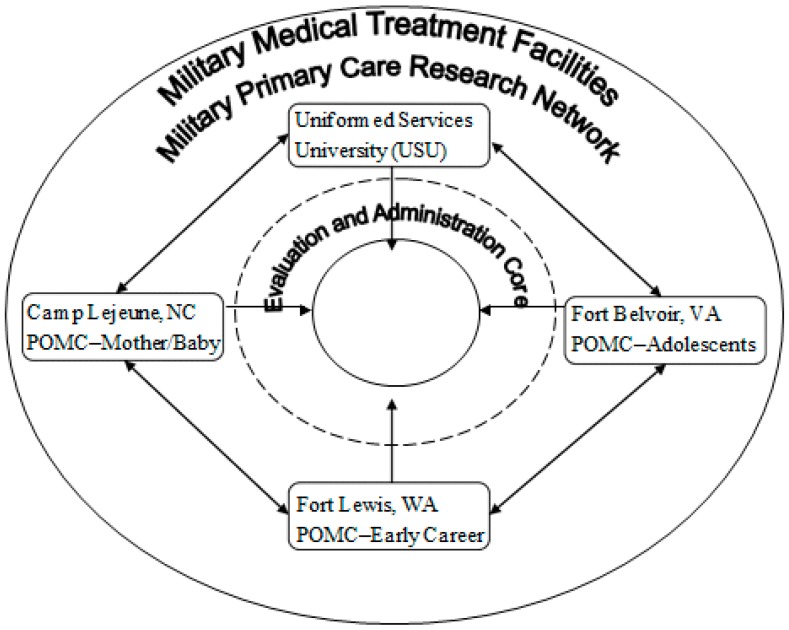
POMC conceptual model.

This paper describes the POMC program, an aggregate of three separate two-group randomized-controlled pilot trials (RCT). Each RCT addresses obesity prevention during a high-risk period: pregnancy/postpartum (Naval Hospital, Camp Lejeune, NC); adolescence (Fort Belvoir Community Hospital, Fort Belvoir, VA, USA); and young adult early career military (Madigan Army Medical Center, Fort Lewis, WA, USA) (Figure 1). Each POMC trial leverages existing DoD healthcare infrastructure to prevent excess weight gain in service members and their families. The efficacy of POMC trials is measured by mitigation of excess weight (Mother/Baby, Adolescent studies) or prevention of excess weight gain (Early Career study). We use percentage of participants who do not gain weight during follow-up and total weight change as markers of our study outcomes. We hypothesize that POMC intervention programs will reduce weight gain beyond the control comparison programs in the period following study participation.

### 2.2. Intervention Rationale

Two types of interventions are used in POMC. A form of counseling called dissonance-based counseling is conducted with pregnant women (POMC—Mother/Baby) and with young adults completing their first term of enlistment in the Army (POMC—Early Career). Our choice to examine the preliminary efficacy of a dissonance-based program for preventing excess weight gain in military populations represents an extension of previous work among civilian young adults and adolescents [18,19,20,21] and promising results of a pilot study (data not shown) we conducted that focused on diet and exercise counseling during routine prenatal and well-child visits to mitigate the trajectory of weight gain in both pregnancy and early infancy. We hypothesize that the dissonance-based approach may be particularly useful for early career service members and pregnant women because it promotes critique of the behaviors leading to excess weight gain and capitalizes on many of the cultural factors inherent in serving in the military (e.g., readiness for duty, maintaining standards so others in your unit can count on you). Additionally, inducing dissonance during pregnancy (a period of transition) may benefit women in mitigating excess weight gain and developing healthy habits by capitalizing on women’s desire to be healthy during the transition to motherhood as pregnancy is a major event and not only affects mothers’ lifestyles but impacts health behaviors [22,23]. 

A total of 120 participants each are recruited for Mother/Baby and Early Career studies. For our Mother/Baby study, we conducted a pilot study with eight pregnant women focusing on diet and exercise counseling during routine prenatal and w ell-child visits to mitigate the trajectory of weight gain in both pregnancy and early infancy. The program was well-received by patients and staff. Enrolled participants attended 100% of the counseling visits. Weight gain during pregnancy was within recommended guidelines for most participants. Infant birth weights were all under the 90th percentile; all but one were between the 25th and 75th percentile. These pilot findings were used to calculate power analyses for one-way within-subjects ANOVA involving different time points. A sample size of 60 per group yielded 80% power for an alpha of 0.05 to detect a group x time interaction of 0.46 standard deviations over 8 visits, meaning that over the course of pregnancy, the weight change in the treatment group differs from weight change in the usual care group by 0.46 standard deviations. If baseline BMI has mean 26 kg/m^2^ and standard deviation 3, this would correspond to a BMI change in the intervention group that is 1.4 BMI points less than the BMI change in the control group.

Power calculations for the Early Career study were based on medium effect sizes observed in preliminary work (d = 0.40‒0.42) with the civilian prevention program from which our Early Career dissonance-based manual was adapted. To calculate sample size estimates, Cohen’s conventions for social science research for a medium effect size (d = 0.25) were chosen for intervention groups and for the repeated measures (time) and the treatment x time interaction. A nondirectional analysis of variance with alpha = 0.05 allowed testing of main and interaction effects of major dependent variables with 80% power using a sample size of 120.

Adolescent dependents are provided interpersonal psychotherapy (IPT) (POMC—Adolescents). The Adolescent study includes 48 participants. Our decision to use IPT as the intervention for our adolescent study is based on years of programmatic work in the area of loss of control (LOC) eating and binge eating among children and adolescents. Of the therapies effective at reducing binge episodes and inducing weight stabilization among obese adults, IPT holds particular promise for the prevention of excess weight gain among adolescents [24,25]. IPT targets the difficult social functioning and stressful events that are associated with LOC and may be highly relevant to adolescent youth from military families who are at elevated risk of difficulties in psychological functioning [14,15,16]. Preliminary pilot research in civilian adolescents indicates that IPT is effective at reducing inappropriate weight gain [24]. We have nearly completed an efficacy trial of IPT for adolescent civilian girls at high risk for adult obesity. Our subsequent efficacy trial comparing IPT to HE [25] (N = 113) was conducted to determine whether IPT is effective at preventing excess weight gain in youth at high-risk for adult obesity who report LOC eating. Median program attendance of girls randomized to IPT was higher than that of girls randomized to HE (11 of 12 sessions [91.7%] *vs.* 10 of 12 sessions [83.3%], *p* = 0.01). Preliminary outcome data (not shown) from 40 adolescents indicated that IPT (but not HE) was associated with participants achieving a normal weight status at 3-year follow-up.

#### 2.2.1. Dissonance-Based Intervention (POMC—Mother/Baby and POMC—Early Career)

Dissonance-based health promotion interventions capitalize on the basic human desire to maintain consistency between one’s words and actions for beneficial outcomes [26]. Dissonance-based counseling-induction activities are designed to promote discrepancy between actions and beliefs regarding food and exercise leading to behavior change [27]. Specifically, POMC—Mother/Baby and POMC—Early Career participants discuss potential adverse effects of obesity, an unhealthy diet, and sedentary behavior. In addition, participants discuss the benefits of maintaining a physically fit body, eating a healthy diet, and engaging in regular physical activity. Discussions in both interventions also center on the costs and benefits as they apply not only to adults, but also to participants’ children. POMC programs focus on weight gain prevention and improvement of health outcomes and psychological well-being, rather than weight loss. Discussions with participants utilize strategies recommended by the American Academy of Family Physicians AIM-HI program [28], encouraging mindful behavior change rather than weight loss. This non-diet approach is used in all group (Early Career) and individual (Mother/Baby) discussions with participants. Conversations not only focus on making sustainable changes but discourage stigmatizing comments about weight, which can increase risk of weight gain [29]. Theoretically, arguing these perspectives allows individuals to make healthier lifestyle choices in the future [26,27]. In an effort to be consistent with the perspectives they argued during the intervention sessions, mothers will make healthier choices to reduce unhealthy weight gain in pregnancy and for their infants. Similarly, young adult participants will make choices that reduce weight gain during their first tour on active duty. The use of strategic self-presentation [30] and verbal commitments from participants to make healthy lifestyle changes helps to maximize dissonance and promote healthy behavior change. Research suggests that people often change their behaviors to conform to the perspectives or behaviors enacted in group settings [31,32]. Such effects putatively emerge because the cognitive dissonance this creates increases the likelihood that participants will act or think in ways that adhere to the perspectives for which they have argued in sessions.

#### 2.2.2. Interpersonal Psychotherapy-Based Intervention (POMC—Adolescents)

The second type of intervention used is interpersonal psychotherapy (IPT) for adolescents (POMC—Adolescents). The program was adapted directly from IPT-Adolescent Skills Training for the prevention of depression [33] and group IPT for binge eating disorder [34]. The IPT program used in the current pilot trial targets loss of control (LOC) eating, a behavior that has been shown to predict excess weight gain [35,36] and partial or full-syndrome binge eating disorder [37,38]; thus, reducing LOC eating may be an important mechanism in the prevention of excess weight gain. Furthermore, preliminary research in civilian adolescents indicates that IPT is effective at reducing inappropriate weight gain [24]. Similar to all IPT programs [39], our adapted IPT follows three stages (initial, middle, and termination) and uses the interpersonal inventory to identify interpersonal problems that might be contributing to or exacerbated by LOC eating. A framework of common problem areas is used to teach interpersonal problem-solving and communication skills and educate youth about risk factors for excessive weight gain. Problematic eating behaviors, such as eating in response to negative affect as opposed to hunger, or feeling a sense of LOC while eating are also reviewed. IPT focuses on psychoeducation and general skill-building that can be applied to different relationships within the framework of three interpersonal problem areas: interpersonal role disputes, role transitions, and interpersonal deficits [24].

Specific adaptations to the IPT process were made for use with adolescent military dependents. For example, clinical scenarios are included that are tailored to the unique experiences of military adolescents. These include frequent relocations, interactions with parents around diet and exercise standards in the military, and coping with parental deployment. Group members are adolescent girls from military families, who understand and are typically able to support each other around these common stressors military families face.

### 2.3. POMC Intervention Commonalities

Each study uses different methods, questionnaires, and schedules for study visits to accommodate the unique needs of the study sample. However, since each study is conducted on a military base, several aspects of study development are similar across sites.

#### 2.3.1. Recruitment

Recruitment includes the following targeted efforts: (1) referrals from physicians and clinic providers; (2) directly from family medicine clinics and advertising within clinics; (3) advertisements in the hospital newsletter and other base periodicals; (4) flyers posted at local base facilities and within the hospital; and (5) mailings to families who receive care at clinics on base or associated satellite clinics.

All recruitment efforts provide a telephone number for interested individuals to call. To avoid any perception of coercion by military rank, a civilian research staff member recruits all participants. Interested participants complete a brief screening over the telephone to determine eligibility. If eligible, a baseline assessment visit is scheduled during which written child assent and/or adult/parent consent take place.

#### 2.3.2. Retention Methods

Participants are contacted by phone prior to each appointment/session. Individuals who miss a session are contacted twice to check-in and provide a brief overview of the missed session topics. Participants who leave the studies for any reason (e.g., due to military orders, relocation, voluntary participant withdrawal, or withdrawal of a participant by a study team member) have their data analyzed by intention-to-treat.

Participants are involved with the POMC program for either 18 months (POMC—Mother/Baby) or 24 months (POMC—Adolescents; POMC—Early Career). These time frames include all intervention visits and 1-year and 2-year follow-ups. Data collection at the 1- and 2-year follow up is facilitated by asking participants to provide permission for investigators to access data from the electronic health record if s/he is otherwise unavailable. All participants in the geographical area are contacted to come to the laboratory to complete follow-up assessments. Electronic records are only accessed if the participant has moved from the area where the study takes place. Data collected from the electronic medical system are obtained by the site principal investigator. For individuals who have not permitted the study team this access or who are no longer in the electronic medical system, self-reported weight and height is collected by telephone or email.

#### 2.3.3. Randomization

Eligible participants are randomly assigned to an intervention or control program in each RCT. All studies use a computer program that creates a randomization string is used to assign participants to one of the two groups in each study. Research administrators at each study site complete the randomization. Participants are notified of the randomization in the informed consent but are not randomized until after baseline screening so outcome assessors remain blind to the group assignation at baseline. An equal number of participants are randomized to intervention and control groups in each study.

#### 2.3.4. Informed Consent

Written informed consent is obtained from all adult participants before data collection begins. Participants are informed of their right to withdraw at any point in the study and the limitations of confidentiality. For the adolescent study, written assent is collected from all youth in addition to written consent from parents/legal guardians. Study sessions are not conducted by patient providers, but as frequently as possible take place in conference rooms within family medicine clinics.

#### 2.3.5. Training of facilitators and supervision

Each RCT uses a standardized training protocol employed in previous prevention trials [24,27]. Investigators on each POMC study conduct initial training sessions for group facilitators and study team members. All sessions are facilitated by a psychologist, registered dietician, nurse, or other designated health care professional. Recordings of study sessions are used to assess implementation fidelity and facilitator competence using reliable and valid methods [20,40,41,42] in Adolescent and Early Career studies. Bi-annual 1-day skill assessments are used in the Mother/Baby to prevent skill drift. All session facilitators are required to read the manual that discusses the conceptual basis for the intervention, review the intervention scripts, and discuss solutions to common problems that may arise during intervention delivery and provide group management skills.

#### 2.3.6. Use of Data

Data collected as part of POMC will provide the foundation for a larger effectiveness trial within the military healthcare system.

### 2.4. Pilot Prevention Programs

#### 2.4.1. Project POMC in Pregnancy and Early Infancy

Our POMC intervention with pregnant women (POMC—Mother/Baby) provides longitudinal counseling on weight-related issues in pregnancy/*post-partum* and early infancy. The approach combines continuity of care, a community-based intervention within a PCMH, and active patient engagement with dissonance-based (referred to as “positive gains” to avoid negativity in pregnancy) counseling during routine prenatal and well-child care visits. Positive gains counseling sessions coincide with existing clinic visits, to enhance PCMH continuity during pregnancy and through the critical first 6 months of life. Studies examining the impact of breastfeeding on mother’s weight are inconclusive as a whole. Yet, multiple professional organizations (e.g., pediatrics, American Congress of Obstetricians and Gynecologists (ACOG)) identify 6 months *post-partum* as the period of time most often recommended for exclusive breastfeeding to prevent excess weight gain in mother and child.

After determination of pregnancy, women are enrolled from either Obstetrics or Family Medicine clinics. Clinical care processes are similar between the two clinics. In keeping with the PCMH model of care, counseling is consistent throughout pregnancy and the *post-partum* period. Low-risk prenatal patients are recruited from maternity care clinics at the Naval Hospital Camp Lejeune, NC and randomized to receive standard care [43] plus positive gains counseling or standard prenatal care. The intervention group receives 7 positive gains counseling sessions in addition to routine prenatal care. Positive gains counseling sessions take place once each trimester and at the 2-week, 2-month, 4-month and 6-month well-child care visits. The control group receives standard prenatal and postpartum care.

##### 2.4.1.1. Inclusion/Exclusion Criteria

All women 18–35 years of age with low-risk pregnancies are eligible to participate. Participants must receive their care within the Military Health System, plan to reside in the study area for at least 18 months, not at elevated risk of complications due to BMI on determination of pregnancy; BMI > 18 and < 30 kg/m^2^) not be actively involved in another weight management program, and speak English. The primary exclusion criteria are current involvement in a structured weight loss program or a medical-risk pregnancy based on VA/DoD Management of Pregnancy Guidelines [43]. As an example, participants are considered at medical risk if they have any underlying medical conditions (e.g., hypertension, thyroid disease) or if they have multiple gestations.

##### 2.4.1.2. Assessment Schedule

Pregnant women and mother-infant dyads are followed from the time of pregnancy verification through the first six months of the infant’s life. POMC-specific assessments are conducted for positive gains counseling and control groups once during each trimester and at the 2-week, 2-month, 4-month and 6-month well-child care visits. Women in the positive gains counseling group also receive counseling administered by medical providers trained by study PI (MS) at each of these visits. Counseling sessions encourage participants to consistently match thoughts, words, and actions for positive choices in dietary intake and physical activity to promote healthy weight trajectories during pregnancy and infancy.

##### 2.4.1.3. Measures

All laboratory and physical data collected are part of normal and routine care processes. It is routine to screen for gestational diabetes at 26–28 weeks. Routine anthropomorphic and physiologic measurements include height, weight blood pressure, and heart rate.

Infant anthropometric measures are assessed at post-natal visits to allow for comparisons of feeding patterns and weight trajectories between children in intervention and control groups. Self-report questionnaires assessing physical/mental health during pregnancy, mood, social support, and self-efficacy for behavior change are administered at each visit (see Table 1 for assessment descriptions and collection time points).

**Table 1:** Assessment schedule.

Measures, by POMC Study	Assessment Point							
	Pregnancy (Trimesters)				Post-Partum Visits			
**POMC—Mother/Baby**	**Consent**	**1**	**2**	**3**	**2 weeks**	**2 months**	**4 months**	**6 months**
Weight Efficacy Lifestyle Questionnaire								
Revised Hopkins Symptom Checklist								
Edinburgh Postnatal Depression Scale								
State-Trait Anxiety Inventory								
Perceived Stress Scale								
Work Climate and Experiences								
Norbeck Social Support Questionnaire								
Physical Fitness Test Results *								
Automated Food Diary (ASA24)								
Physical Assessments								
Height and Weight (BMI)	Mother: each visit				Mother/child: each visit			
Blood pressure and heart rate	Mother: each visit				Mother/child: each visit			
Waist circumference	Mother: each visit							
Child head circumference (cm)					Child: each visit			
**POMC—Adolescents**	**Screening/Consent**		**Post-Program**		**1-Year Follow-Up**		**2-Year Follow-Up**	
Eating Disorder Examination								
Schedule for Affective Disorders and Schizophrenia for School-Age Children								
Social Adjustment Scale—Self-Report								
Family Adaptation and Cohesion Evaluation Scales Inventory								
Beck Depression Inventory								
State-Trait Anxiety Inventory for Children—A trait scale								
Life Events and Coping Inventory								
Eating Disorder Diagnostic Scale								
Treatment Acceptability Questionnaire								
Inventory of Parent and Peer Attachment								
Family Resilience Assessment Scale								
Perceived Stress Scale								
The Emotional Eating Scale—Dapted for Children								
Physical Assessments								
Height and Weight								
Blood Pressure								
Waist Circumference								
Blood Draw								
Parent Questionnaires:								
Parent Stress Index—Short Form								
Child Behavior Checklist								
**POMC—Early Career**	**Screening/Consent**		**Post-Program**		**1-Year Follow-Up**		**2-Year Follow-Up**	
Questionnaire on Eating and Weight Pattern—Revised								
Preference for Consistency Scale								
International Physical Activity Questionnaire								
Block Food Frequency Questionnaire								
Eating Disorder Diagnostic Scale								
Beck Depression Inventory-II								
Emotional Eating Scale								
Physical Assessments								
Height and Weight								
Blood Pressure								
Neck, Waist, Hip Circumference								

Note: * Active duty only.

##### 2.4.1.4. Intervention Groups

After pregnancy verification, participants are randomly assigned to usual care or positive gains counseling groups.

##### 2.4.1.5. Usual Care

The usual care group receives routine prenatal care in accordance with accepted guidelines [43]. This includes regular clinic visits and screening laboratories for cystic fibrosis, trisomy 21, gestational diabetes, anemia, hepatitis, HIV, syphilis, gonorrhea, chlamydia, cervical cancer screening, urinalysis and culture and screening for group B streptococcal infections. Weight, blood pressure, heart rate, and fundal height are routinely assessed at each clinic visit. Similarly, infant height, weight, head circumference and vital signs are assessed at each well-child visit. During routine well-child care visits, developmental milestones are reviewed, a thorough history is taken and physical examination is performed. Age-appropriate immunizations are provided as is parental anticipatory guidance, including the importance of breastfeeding. Participants in both groups receive guidance from their primary care provider. This includes general advice about feeding and infant care. Providers deliver this anticipatory guidance in their usual fashion with no external cues or counseling provided by the research team. All encounters are documented in the patient’s electronic medical record.

##### 2.4.1.6. Positive Gains Counseling

Once each trimester and at 2-week, 2-month, 4-month and 6-month well-child visits (7 positive gains counseling sessions total), women in the intervention group receive counseling that focused on the benefits of being physically fit, eating healthy foods, and regular exercise, and the costs of obesity, a high-fat/high-sugar diet, and sedentary behavior. Specific counseling (whenever possible, provided by the same counselor throughout pregnancy and early infancy) focuses on positive-gain-based cognitive strategies to promote breastfeeding, recognize infant satiety cues, and promote healthy food choices. Each counseling session has a different topic related to issues specific to each stage of pregnancy/infancy (e.g., preparing for birth, initiating and maintaining breastfeeding, introducing solid foods).

We propose that positive gains counseling has particular utility for first-time mothers and military members or spouses who don’t have family nearby. By incorporating counseling sessions with pre-existing clinic visits, participants receive additional social and emotional support during pregnancy and after they give birth. We believe that discussing issues that impact the long-term health and well-being of mother and infant encourages continued communication about weight and nutrition-related issues, strengthening the PCMH-based patient-provider team relationship.

#### 2.4.2. Project POMC in Adolescents

Since the adolescent daughters of military personnel report high rates of disordered eating [17], and LOC eating has been shown to predict excess weight gain, exacerbated metabolic functioning, and partial and full syndrome eating disorders [35,37,38,44,45], we hypothesize that IPT will decrease LOC eating and reduce these adverse outcomes. A secondary hypothesis of this study is that girls in the IPT group who maintain their weight or experience weight loss will demonstrate an improvement in components of the metabolic syndrome at follow-up visits.

This pilot study involves a RCT to pilot the implementation of IPT for the prevention of inappropriate weight gain in adolescent female military dependents. Our adapted IPT aims to slow the trajectory of weight gain among female dependents at high-risk for adult obesity by virtue of elevated BMI percentile(≥85th percentile) and current or past (within 3 months) LOC.

IPT will be compared to a health education control to assess the acceptability and feasibility of the prevention program. Further, POMC in Adolescents will assess whether IPT influences body weight gain trajectories and prevents worsening disordered eating and metabolic functioning among female military dependents at heightened risk for unhealthy weight gain.

##### 2.4.2.1. Inclusion/Exclusion Criteria

Female military dependents, age 12–17 years, at high risk for excess weight gain (BMI ≥ 85th percentile) who report lifetime LOC eating or at least two current indicators of LOC eating (e.g., eating in response to negative affect, feelings of guilt or shame around eating) [46], are recruited from the Family Medicine Clinic at Fort Belvoir Community Hospital. Participants are excluded for presence of a chronic major medical illness (e.g., renal, hepatic, gastrointestinal, endocrinologic), documented, obesity-related medical complication requiring a more intensive intervention approach, psychiatric conditions (e.g., psychosis, suicidality) that would impede program participation, current weight loss treatment, current weight-affecting medication usage, and pregnancy (current or recent) or current breast-feeding.

##### 2.4.2.2. Assessment Schedule

Assessments for the adolescent participants are conducted four times, once each at baseline, post-prevention (end of group program), and 1-year and 2-year follow-ups.

##### 2.4.2.3. Measures

For each assessment, participants are asked to fast overnight prior for the measurement of height and weight and blood withdrawal. At all assessments, waist circumference and blood pressure are measured, and fasting phlebotomy is conducted to assess indicators of obesity and metabolic syndrome, such as HDL cholesterol, triglycerides, glucose, insulin, and hemoglobin A1c, and questionnaires completed. Participants and the accompanying parent or guardian are asked to complete questionnaires to assess perception of their own and their child’s mental and physical wellness, and adolescent participants complete interviews regarding their eating behaviors and psychiatric symptoms, such as depression and anxiety (see Table 1).

##### 2.4.2.4. Intervention Groups

After baseline screening, participants are randomly assigned to receive IPT or health education control, stratified by the presence of reported LOC.

##### 2.4.2.5. Health Education

The health education control group receives a workbook on how to live a healthier life, discussing topics unrelated to eating. Participants complete 4 monthly 90-minute group meetings during after school hours to discuss the information presented in the workbook. The workbook is based upon the book *7 Habits of Highly Effective Teens* by Sean Covey [47]. The workbook will serve as a guide for teens to improve their self-image, build friendships, resist peer pressure, and engage in goal-setting.

##### 2.4.2.6. IPT for the Prevention of Excess Weight Gain

Our adapted IPT [24,34,48] involves one initial 1.5 hour individual session, and 12 weekly 90-minute group sessions that take place after school hours. During the pre-group individual session, the leaders meet with each participant to begin establishing rapport, provide a framework for the group intervention, and conduct an interpersonal inventory. The interpersonal inventory is a semi-structured clinical interview that identifies the strengths and problems in the adolescent’s important relationships and self-identified interpersonal goals of the adolescent. This information is used by the group leader to link problems in the adolescent’s interpersonal relationships to her LOC eating and other disturbed eating attitudes or behaviors. Collaboratively, 1–2 interpersonally-related goals are set that are worked on, and revised as necessary, throughout the 12 weeks of group.

The initial phase of the intervention (Sessions 1–3) involves psychoeducation, including defining prevention, identifying feelings, education about the predictors of excess weight gain, outlining the 3 interpersonal problem areas, and discussing the link between feelings and interpersonal interactions. Various communication and interpersonal strategies are taught, such as using “I statements” and finding the right time to have a conversation. Group members practice these skills, first using games and hypothetical situations and then using role-plays. The middle phase of the intervention (Sessions 4–9) focuses on how these communication and interpersonal strategies can be applied to different, specific relationships in the adolescents’ lives, particularly relationships that are linked to LOC and emotionally-induced eating. During the termination phase (Sessions 10–12), the group reviews improvements in LOC eating and related symptoms, with a focus on identifying possible warning signs of a return or increase in problematic eating and knowing when to seek help. Group members are encouraged to continue the interpersonal work on their own.

IPT is particularly relevant to the unique stressors burdening many children of military personnel. These often include multiple moves (sometimes every 1–2 years, including internationally) and making new friends and coping with the deployment of a parent. Although military adolescents typically attend school in the civilian community, the group program provides the opportunity for adolescents from military families to garner support from other military adolescent girls, with whom their experiences and challenges resonate.

#### 2.4.3. Project POMC in Early Career Military

The period following basic training is a time when young adults are at high-risk for gaining rebound weight following basic training if daily physical activity decreases. Compared to recruits without a predisposition to overweight or obesity, young adults with a personal or family history of excess weight are at elevated risk of rebound weight regain following weight loss during intense military training [49]. The POMC—Early Career prevention trial compares a brief, 6-week, dissonance-based group program (Fit4Duty) designed to reduce unhealthy weight gain to a health education control among early career enlistees at-risk for obesity. The Fit4Duty program is an adaptation for the military of an existing civilian obesity prevention program, *Project Health* [18,19,20], which reduced obesity onset by 50% in healthy civilian young adults.

The goal of Project Fit4Duty is to reduce excessive weight gain beyond the health education program in the two years following study participation among at-risk early career military.

##### 2.4.3.1. Inclusion/Exclusion Criteria

Participants must be 18 years or older, have proficiency in the English language, not be scheduled for re-assignment within 2 months of starting the program, not at immediate risk of discharge due to weight (BMI > 18.5 but ≤ 32), and have a risk factor for excess weight gain. Risk for excess weight gain is defined by either a personal or family history of overweight or obesity. Participants are excluded for major medical illnesses (e.g., heart attack, cancer), conditions that affect body weight (e.g., thyroid disease) and planned deployment or off-site training within two months of starting the program.

##### 2.4.3.2. Assessment Schedule

The study includes four assessments: baseline, 6-weeks (post-programs), and 1-year and 2-year follow-ups. Participants randomized to Project Fit4Duty meet weekly for six group sessions. Participants randomized to the health education program meet twice over six weeks.

##### 2.4.3.3. Measures

All participants complete height, weight, blood pressure, neck, waist, and hip measurements and self-report instruments at each assessment and complete questionnaires (refer to Table 1).

##### 2.4.3.4. Intervention Groups

After baseline screening, participants are randomized to complete Project Fit4Duty or health education.

##### 2.4.3.5. Health Education

The video education series, *Healthy Eating: A Guide to Nutrition* [50], is delivered over 2 weekly 1-hour sessions in small groups of 6–10 participants. Videos focus on basic nutrition, nutrition and weight management, nutrition for sports and exercise, and food safety/disease prevention.

##### 2.4.3.6. Project Fit4Duty

Project Fit4Duty involves six weekly 1 hour sessions with 6–10 participants facilitated by a trained counselor. Fit4Duty uses dissonance-based counseling-induction activities derived from the civilian *Project Health* program, adapted for military personnel. Each participant develops an individualized plan for dietary and activity changes with the intention of balancing caloric intake and expenditure long-term. Research suggests that people often change their behaviors to conform to the perspectives or behaviors enacted in group settings [31,32]. The intervention also uses strategic self-presentation [30] wherein participants make verbal commitments to making healthy lifestyle changes in a group setting. Session activities focus on costs of obesity, an unhealthy diet, and sedentary behavior and the benefits of fitness, a healthy diet, and regular exercise specific to early career military personnel in late adolescence/young adulthood through a series of verbal, written, and behavioral exercises. For example, individuals are encouraged to evaluate the costs of obesity in terms of risk to self, risk to others, and impact on ability to serve effectively as a unit.

The Fit4Duty program is designed especially for soldiers who are intrinsically motivated to prevent weight gain in order to continue a career in the military. Whereas existing programs available in the military tend to focus on weight management using nutrition and fitness education in either one-on-one or group settings, Fit4Duty uses strategies and techniques successful at preventing weight gain among young adult civilians and focuses on prevention rather than management. The pilot program offers soldiers an additional resource if they are concerned about ‘making weight,’ providing options to them before they exceed military weight standards and face professional (e.g., overlooked for promotion) or personal (e.g., stigma) penalties.

## 3. Results and Discussion

The POMC program is a comprehensive obesity prevention research program integrated within PCMHs in Military Training Facilities. POMC is designed to prevent excess weight gain and improve the health and readiness of service members and their families while maintaining continuity of care. All study sites employ the PCMH model of care, which emphasizes continuity and longitudinal provider-patient relationships. We believe that implementing weight gain prevention programs within the PCMH may support behavior change, in part, by utilizing established relationships between patients and the provider care team.

Though the population impact of our POMC model is currently unclear since we have not completed our pilot trials or subsequent larger-scale effectiveness trials, we have found that developing prevention programs in collaboration with specific populations and social systems results in programs that are uniquely tailored to the needs of that population and are far more likely to be disseminated within that system [51]. Given the growing rate of overweight and obesity among active duty personnel and dependents, results from POMC studies have the opportunity to impact a substantial number of people who are at-risk for excess weight gain in the military healthcare system. Overweight and obesity disproportionately affect some military personnel more than others. Service members who are male, older, and African American or Hispanic are at higher risk [52,53]. With over one-third of the military comprised of self-identified racial (e.g., African American) and ethnic (*i.e.*, Hispanic) service members [54], results from our studies may be particularly beneficial for these under-represented individuals. While military women are far less likely to be overweight (34.4%) or obese (6.4%) compared to their male counterparts (54.2%; 13.5%), females comprise 15% of the military population [3]. Therefore, study outcomes are likely to inform not only military-specific concerns, but also those of under-represented racial and ethnic groups. Such data will inform the development of culturally tailored prevention programs.

Use of the MPCRN to conduct the project also facilitates future research and promotes immediate dissemination of clinical findings. It is our belief that PCMH are ideally positioned to provide early recognition, education, and intervention in the continuing obesity epidemic. Prevention through the PCMH Family uses an existing network of Family Practice physicians who report the need to address obesity prevention and the need for improved prevention programs [55,56]. Initiatives such as Performance Triad continue to encourage improved sleep, activity, and nutrition (e.g., health behaviors) and we hope the presence of programs such as POMC will provide another option for patients who express an interest in preventing weight gain. POMC programs address barriers to behavior change, including limited access and availability of healthy food options, eating healthy on a budget, preventing overuse injuries, active coping to manage job-related stress, and obstacles that affect military populations such as weight stigma and disordered eating to “make weight” [54,57]. As more and more physicians are recommending behavioral modification practices [56], military family physicians practicing within the PCMH model are in an ideal position to help stem the rise in obesity by using long-term solutions, such as the behavior modification programs that we are proposing in our studies. In addition, this project will further strengthen the existing MPCRN infrastructure allowing for an increased capacity to study weight-related health behaviors.

Further, POMC applies interventions that have been used with civilian samples [24,27], tailored specifically for military personnel and beneficiaries. The use of a trans-disciplinary research team—comprised of primary care physicians, health psychologists, public health experts, and social scientists—working together within PCMHs positions the POMC research program to disseminate study findings in many of or all U.S. Military Training Facilities in order to move the field of obesity prevention-focused research forward.

## 4. Conclusions

At the completion of this project, we will have examined the feasibility and acceptability of three obesity prevention programs (POMC—Mother/Baby, POMC—Adolescent, and POMC—Early Career) at three MPCRN sites (Camp Lejeune, Fort Belvoir, Fort Lewis). The next step will involve carrying out fully-powered trials. If successful, each of these programs has the potential to be implemented in PCMH settings within both the military and civilian communities. Regardless of the study findings, the POMC collaboration will contribute to the sparse literature on eating and weight problems within the U.S. Military. Understanding the lifestyle and social behaviors of service members and their families will help to better promote strategies for mitigating obesity in military communities and on a larger public scale.

## References

[1] Ogden C., Carroll M., Kit B., Flegal K. (2014). Prevalence of childhood and adult obesity in the United States, 2011–2012. J. Amer. Med. Assoc..

[2] Eilerman P.A., Herzog C.M., Luce B.K., Chao S.Y., Walker S.M., Zarzabal L.A., Carnahan D.H. (2014). A comparison of obesity prevalence: Military health system and United States populations, 2009–2012. Mil. Med..

[3] Barlas F.M., Higgins W.B., Pflieger J.C., Diecker K. (2013). 2011 Department of Defense Health Related Behaviors Survey of Active Duty Military Personnel.

[4] Registry M.B. Military Brat Survey Results. http://www.militarybrat.com/surveyresults2b.cfm.

[5] Raghuveer G. (2010). Lifetime cardiovascular risk of childhood obesity. Amer. J. Clin. Nutr..

[6] Weiss R., Dziura J., Burgert T.S., Tamborlane W.V., Taksali S.E., Yeckel C.W., Allen K., Lopes M., Savoye M., Morrison J. (2004). Obesity and the metabolic syndrome in children and adolescents. N. Engl. J. Med..

[7] Dietz W.H. (1998). Health consequences of obesity in youth: Childhood predictors of adult disease. Pediatrics.

[8] Freedman D., Khan L., Serdula M., Dietz W., Srinivasan S., Berenson G. (2004). Inter-relationships among childhood BMI, childhood height, and adult obesity: The bogalusa heart study. Int. J. Obes. Relat. Metab. Disord..

[9] Field A.E., Cook N.R., Gillman M.W. (2005). Weight status in childhood as a predictor of becoming overweight or hypertensive in early adulthood. Obes. Res..

[10] Dall T.M., Zhang Y., Chen Y.J., Wagner R.C., Hogan P.F., Fagan N.K., Olaiya S.T., Tornberg D.N. (2007). Cost associated with being overweight and with obesity, high alcohol consumption, and tobacco use within the Military Health System’s TRICARE prime-enrolled population. Amer. J. Health Promot..

[11] Department of Veterans Affairs/Department of Defense (VA/DoD) (2014). VA/DoD Clinical Practice Guideline for Screening and Management of Overweight and Obesity. http://www.healthquality.va.gov/guidelines/CD/obesity/VADoDCPGManagementOfOverweightAndObesityFinal.pdf.

[12] Maguen S., Madden E., Cohen B., Bertenthal D., Neylan T., Talbot L., Grunfeld C., Seal K. (2013). The relationship between body mass index and mental health among Iraq and Afghanistan veterans. J. Gen. Intern. Med..

[13] Littman A.J., Boyko E.J., McDonell M.B., Fihn S.D. (2012). Evaluation of a weight management program for veterans. Prev. Chronic. Dis..

[14] Rosen L.N., Teitelbaum J.M., Westhuis D.J. (1993). Children’s reactions to the desert storm deployment: Initial findings from a survey of army families. Mil. Med..

[15] Peebles-Kleiger M.J., Kleiger J.H. (1994). Re-integration stress for desert storm families: Wartime deployments and family trauma. J. Trauma Stress.

[16] Jensen P.S., Martin D., Watanabe H. (1996). Children’s response to parental separation during operation desert storm. J. Amer. Acad. Child Adolesc. Psy..

[17] Waasdorp C.E., Caboot J.B., Robinson C.A., Abraham A.A., Adelman W.P. (2007). Screening military dependent adolescent females for disordered eating. Mil. Med..

[18] Stice E., Shaw H., Burton E., Wade E. (2006). Dissonance and healthy weight eating disorder prevention programs: A randomized efficacy trial. J. Consult. Clin. Psychol..

[19] Stice E., Marti C.N., Spoor S., Presnell K., Shaw H. (2008). Dissonance and healthy weight eating disorder prevention programs: Long-term effects from a randomized efficacy trial. J. Consult. Clin. Psychol..

[20] Stice E., Rohde P., Gau J., Shaw H. (2009). An effectiveness trial of a dissonance-based eating disorder prevention program for high-risk adolescent girls. J. Consult. Clin. Psychol..

[21] Stice E., Rohde P., Shaw H, Gau J. (2011). An effectiveness trial of a selected dissonance-based eating disorder prevention program for female high school school students: long-term effects. J. Consult. Clin. Psychol..

[22] Olson C.M. (2005). Tracking of food choices across the transition to motherhood. J. Nutr. Educ. Behav..

[23] Rhodes R.E., Symons Downs D., Bellows-Riecken, Allerton L.T., Rutherfode G.P. (2008). Delivering inactivity: A review of physical activity and the transition to motherhood. Exercise and Women’s Health: New Research.

[24] Tanofsky-Kraff M., Wilfley D.E., Young J.F., Mufson L., Yanovski S.Z., Glasofer D.R., Salaita C.G., Schvey N.A. (2010). A pilot study of interpersonal psychotherapy for preventing excess weight gain in adolescent girls at-risk for obesity. Int. J. Eat. Disord..

[25] Tanofsky-Kraff M., Shomaker L.B., Wilfley D.E., Young J.F., Sbrocco T., Stephens M., Ranzenhofer L.M., Elliott C., Brady S., Radin R.M. (2014). Targeted prevention of excess weight gain and eating disorders in high-risk adolescent girls: A randomized controlled trial. Amer. J. Clin. Nutr..

[26] Festinger L. (1957). A Theory of Cognitive Dissonance.

[27] Stice E., Shaw H., Becker C.B., Rohde P. (2008). Dissonance-based interventions for the prevention of eating disorders: Using persuasion principles to promote health. Prev. Sci..

[28] American Academy of Family Physicians AIM-HI Practice Manual 2013. http://www.aafp.org/dam/AAFP/documents/patient_care/fitness/AIMPracticeManual.pdf.

[29] Sutin A.R., Terracciano A. (2013). Perceived weight discrimination and obesity. PLoS One.

[30] Cialdini R.B., Goldstein N.J. (2004). Social influence: Compliance and conformity. Annu. Rev. Clin. Psychol..

[31] Killen J.D. (1985). Prevention of adolescent tobacco smoking: The social pressure resistance training approach. J. Child Psychol. Psychiat..

[32] Leake R., Friend R., Wadhwa N. (1999). Improving adjustment to chronic illness through strategic self-presentation: An experimental study on a renal dialysis unit. Health Psychol..

[33] Young J., Mufson L. (2003). Manual for Interpersonal Psychotherapy-Adolescent Skills Training (IPT-AST).

[34] Wilfley D., MacKenzie K., Welch R., Ayres V., Weissman M. (2000). Interpersonal Psychotherapy for Group.

[35] Sonneville K.R., Horton N.J., Micali N., Crosby R.D., Swanson S., Solmi F., Field A. (2013). Longitudinal associations between binge eating and overeating and adverse outcomes among adolescents and young adults: Does loss of control matter?. JAMA Pediatr..

[36] Tanofsky-Kraff M., Yanovski S., Schvey N., Olsen C., Gustafson J., Yanovski J. (2009). A prospective study of loss of control eating for body weight gain in children at high risk for adult obesity. Int. J. Eat. Disord..

[37] Hilbert A., Hartmann A., Czaja J., Schoebi D. (2013). Natural course of preadolescent loss of control eating. J. Abnorm. Psychol..

[38] Tanofsky-Kraff M., Shomaker L.B., Olsen C., Roza C.A., Wolkoff L.E., Columbo K.M., Raciti G., Zocca J.M., Wilfley D.E., Yanovski S.Z. (2011). A prospective study of pediatric loss of control eating and psychological outcomes. J. Abnorm. Psychol..

[39] Weissman M., Markowitz J., Klerman G. (2000). Comprehensive Guide to Interpersonal Psychotherapy.

[40] Wilfley D.E., Welch R.R., Stein R.I., Spurrell E.B., Cohen L.R., Saelens B.E., Dounchis J.Z., Frank M.A., Wiseman C.V., Matt G.E. (2002). A randomized comparison of group cognitive-behavioral therapy and group interpersonal psychotherapy for the treatment of overweight individuals with binge-eating disorder. Arch. Gen. Psychiat..

[41] Loeb K.L., Wilson G.T., Labouvie E., Pratt E.M., Hayaki J., Walsh B.T., Agras W.S., Fairburn C.G. (2005). Therapeutic alliance and treatment adherence in two interventions for bulimia nervosa: A study of process and outcome. J. Consult. Clin. Psychol..

[42] Stice E., Sysko R., Roberto C.A., Allison S. (2010). Are dietary restraint scales valid measures of dietary restriction? Additional objective behavioral and biological data suggest not. Appetite.

[43] Department of Veterans Affairs & Department of Defense (2009). Va/DoD Clinical Practice Guideline for Management of Pregnancy.

[44] Tanofsky-Kraff M., Cohen M.L., Yanovski S.Z., Cox C., Theim K.R., Keil M., Reynolds J.C., Yanovski J.A. (2006). A prospective study of psychological predictors of body fat gain among children at high risk for adult obesity. Pediatrics.

[45] Tanofsky-Kraff M., Shomaker L., Stern E.A., Miller R., Sebring N., Dellavalle D., Yanovski S.Z., Hubbard V.S., Yanovski J.A. (2012). Children’s binge eating and development of metabolic syndrome. Int. J. Obes..

[46] Tanofsky-Kraff M., Goossens L., Eddy K.T., Ringham R., Goldschmidt A., Yanovski S.Z., Braet C., Marcus M.D., Wilfley D.E., Olsen C. (2007). A multisite investigation of binge eating behaviors in children and adolescents. J. Consult. Clin. Psychol..

[47] Covey S. (1998). 7 Habits of Highly Effective Teens.

[48] Young J.F., Mufson L., Davies M. (2006). Efficacy of interpersonal psychotherapy-adolescent skills training: An indicated preventive intervention for depression. J. Child. Psychol. Psychiat..

[49] Sumithran P., Proietto J. (2013). The defence of body weight: A physiological basis for weight regain after weight loss. Clin. Sci..

[50] Meridian Education (2011). Healthy Eating: A Guide to Nutrition.

[51] Becker C.B., Stice E., Shaw H., Woda S. (2009). Use of empirically supported interventions for psychopathology: Can the participatory approach move us beyond the research-to-practice gap?. Behav. Res. Ther..

[52] Smith T.J., Marriott B.P., Dotson L., Bathalon G.P., Funderburk L., White A., Hadden L., Young A.J. (2012). Overweight and obesity in military personnel: Sociodemographic predictors. J. Obes..

[53] Sanderson P.W., Clemes S.A., Biddle S.J. (2011). The correlates and treatment of obesity in military populations: A systematic review. Obes. Facts.

[54] Tanofsky-Kraff M., Sbrocco T., Theim K.R., Cohen L.A., Mackey E.R., Stice E., Henderson J.L., McCreight S.J., Bryant E.J., Stephens M.B. (2013). Obesity and the U.S. military family. J. Obes..

[55] Loomis G.A., Connolly K.P., Clinch C.R., Djuric D.A. (2001). Attitudes and practices of military family physicians regarding obesity. Mil. Med..

[56] Warner C.H., Warner C.M., Morganstein J., Appenzeller G.N., Rachal J, Grieger T. (2008). Military family physician attitudes toward treating obesity. Mil. Med..

[57] Garber A.K., Boyer C.B., Pollack L.M., Chang Y.J., Shafer M.A. (2008). Body mass index and disordered eating behaviors are associated with weight dissatisfaction in adolescent and young adult female military recruits. Mil. Med..

